# Accuracy of Computer-Assisted Flapless Implant Placement by Means of Mucosa-Supported Templates in Complete-Arch Restorations: A Systematic Review

**DOI:** 10.3390/ma15041462

**Published:** 2022-02-16

**Authors:** Paolo Carosi, Claudia Lorenzi, Fabrizio Lio, Pierluigi Cardelli, Alessandro Pinto, Andrea Laureti, Alessandro Pozzi

**Affiliations:** 1 Department of Chemical Science and Technologies, Materials for Health, Environment and Energy—Dentistry, University of Rome “Tor Vergata”, 00037 Rome, Italy; paolo.carosi@alumni.uniroma2.eu (P.C.); fabrizio.lio@alumni.uniroma2.eu (F.L.); pintosmile25@gmail.com (A.P.); alaureti97@gmail.com (A.L.); 2 Department of Clinical Sciences and Translational Medicine, School of Dentistry, University of Rome “Tor Vergata”, 00037 Rome, Italy; pieluigi.cardelli@uniroma2.it; 3 Goldstein Center for Esthetic and Implant Dentistry, Department of Restorative Sciences, Augusta University, Augusta, GA 30912, USA; apozzi@augusta.edu

**Keywords:** computer-guided-surgery, immediate loading, digital workflow, complete-arch

## Abstract

The aim of this study was to systematically review the current scientific literature regarding the accuracy of fully guided flapless implant positioning for complete-arch rehabilitations in edentulous patients and to assess if there was any statistically significant correlation between linear deviation at shoulder point, at apex point and angular deviation. The electronic and manual literature search of clinical studies was carried out using specified indexing terms. A total of 13 studies were eligible for qualitative analysis and 277 edentulous patients were rehabilitated with 1556 implants patients by means of fully guided mucosa-supported template-assisted flapless surgery. Angular deviation was 3.42° (95% CI 2.82–4.03), linear deviation at shoulder point 1.23 mm (95% CI 0.97–1.49) and linear deviation at apex point 1.46 mm (95% CI 1.17–1.74). No statistically significant correlations were found between the linear and angular deviations. A statistically significant correlation was found between the two linear deviations (correlation coefficient 0.91) that can be summarized by the regression equation y = 0.03080 + 0.8254x. Computer-assisted flapless implant placement by means of mucosa-supported templates in complete arch restorations can be considered a reliable and predictable treatment choice despite the potential effects that flapless approach could bring to the overall treatment.

## 1. Introduction

Prosthetically driven dental implant placement in complete-arch restorations positively contributed to achieve both functionally and aesthetic results [1,2]. Continuous development of digital technologies led to a more comprehensive pre-surgical evaluation of the implant recipient site, avoiding bad results during follow-up period [3,4]. The introduction of the cone-beam computed tomography (CBCT) scan, the ongoing development of intraoral optical scanners (IOS) and modeling softwires allowed the clinician to digitally navigate through the three-dimensional (3D) bone and soft tissue architecture of the virtual patients into implant-planning software [5,6]. Implant positioning was more predictable and accurate since template-assisted surgery was introduced. The surgical templates were realized in order to fully guide the implant placement through metal sleeves that rigidly guide the dedicated drills. The clinicians were not allowed to change the pathway of the drills unless a freehand implant positioning was performed [7]. It is crucial to stabilize the surgical templates before the start of the bone drilling [8]. The surgical templates can be tooth-, mucosa- or bone-supported. Several studies reported that tooth-supported surgical templates led to a more accurate implants placement if compared to mucosa- or bone-supported templates [9]. However, in case of fully edentulous patients, the mucosa- or bone-supported templates were used and fixed onto the edentulous jaws by means of anchor pins. The stabilization of bone-supported templates required to open a full-thickness mucoperiosteal flap. The mucosa-supported templates can be fixed to the edentulous jaw without raising a flap and implant positioning can be performed flapless, reducing patient discomfort, surgical time and healing period. In reality, the mucosal resilience and the muscular tensions may interfere with the seating of the surgical templates, resulting in a less accurate implant positioning if flapless surgery is performed. However, the flapless approach on complete edentulous arches implant-supported rehabilitation with computer-guided surgical implant placement via mucosa-supported templates could decrease the overall treatment morbidity, reducing costs, time and pain for the patients [10,11].

**Figure 1:**
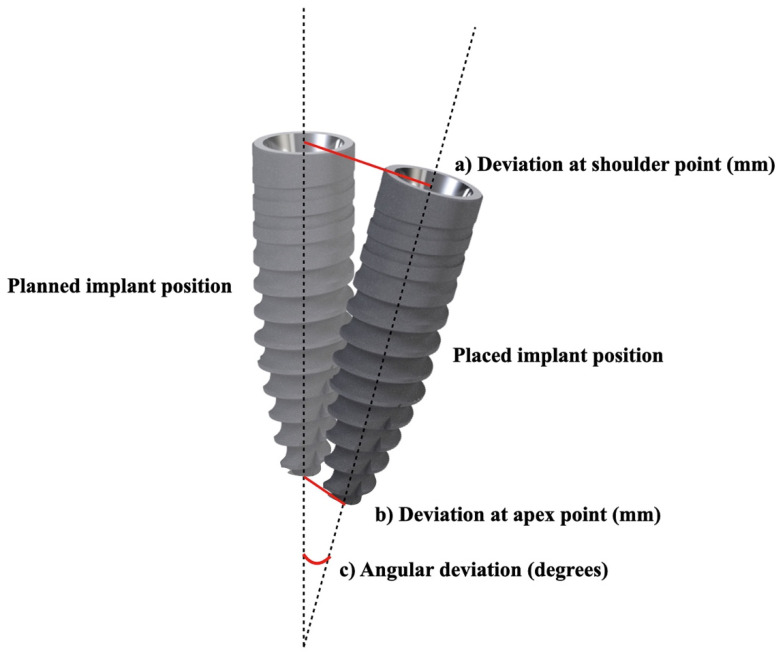
Deviation of placed implants coordinates from 3D planned implants coordinates (a: linear deviation at shoulder point; b: linear deviation at apex point; c: angular deviation).

The accuracy of implant positioning depends on the quantity of linear and angular deviations occurring between the implant’s planned position and the effective implant position [12] (Figure 1). In addition, despite the type of surgical template used, it seems that every step in the digital planning may badly affect the effective implant position [13]. The aim of this study was to review the literature in order to assess the accuracy of implant placement by means of static template mucosa-supported computer guided flapless surgery in complete-arch rehabilitations and to evaluate possible correlations between angular and linear deviations.

## 2. Materials and Methods

This systematic review was conducted in accordance with the PRISMA (preferred reporting items for systematic review and meta-analyses) statement of 1996 and revised in 2020 [14]. The focus was expressed in PICOS (population, intervention, comparison, outcomes and study design) format: P, edentulous patients who received complete-arch rehabilitations; I, implants positioned by means of static mucosa-supported template assisted flapless surgery; C, positioned implants coordinate from preplanned implants coordinates; O, angular deviation, linear deviation at shoulder and at apex points; S, randomized clinical trials (RCTs) and prospective cohort studies. The inclusion and exclusion criteria were defined before the start of the study. The included studies had to be clinical trials published in English, based on human subjects and have at least 10 patients. The research subject had to evaluate linear and angular deviation at shoulder and apex from the preplanned implant coordinates in complete-arch rehabilitations. All studies analyzed have been published between 1 January 2010 and 30 June 2021.

Studies were not included in cases when the same authors published the same data in a different paper or in systematic reviews, commentaries and letters to the editor, case reports or series, in vitro studies, studies in animal models. The text analysis and the data collection were performed in a double-blind way and any disagreement were solved by the authors.

### 2.1. Types of Interventions

The analyzed studies were randomized controlled clinical trials or prospective trials. Only studies that investigated the accuracy of implant placement by means of computer-guided flapless surgery with mucosa-supported static templates were included in this review.

### 2.2. Outcome Measures

The primary outcome was to assess the angular and the linear deviations at shoulder and at apex point, between planned and placed implant by means of static mucosa-supported template in complete-arch rehabilitations with flapless surgery. The secondary outcome was to assess if there was any statistically significant correlation between linear deviation at shoulder point, at apex point and angular deviation. All the collected and analyzed data were measured before intervention.

### 2.3. Search Strategy

Electronic research was performed involving different databases (PubMed, Medline, Embase and Cochrane Library). The following combination of words was used: “surgery AND computed-assisted OR computer-assisted OR therapy computed-assisted OR computer-aided design AND dental implants OR oral implants AND full-arch OR complete-arch OR dental prosthesis OR implant-supported OR implant treatment OR computed guided OR guided surgery”. In addition, bibliographies of reviews were analyzed and cross-checked. The following combination of words was used for the search in the Cochrane database: computer assisted surgery AND dentistry. Moreover, bibliographies of relevant systematic reviews were analyzed to cross-check the data. The authors of the eligible manuscripts were contacted in case further information or data were needed. 

### 2.4. Selection Criteria and Data Extraction

A three-stage examination of the potentially included studies was performed by two reviewers (Claudia Lorenzi and Paolo Carosi). The first stage involved the title analysis. Then, the abstracts were analyzed and only the selected studies were included for the full-text reading. Data results are available at Supplementary Files.

### 2.5. Risk of Bias

The quality of the included studies was assessed independently by the reviewers (Claudia Lorenzi and Paolo Carosi) using the Cochrane Collaboration’s tool for assessing risk of bias [15] in randomized trials. A study with low risk of bias provided all the required information about the investigated parameters. A study with moderate risk did not provide all the information required to fulfill the review process. A study with more than 2 missing parameters was classified as high risk of bias.

Additionally, all of the non-randomized clinical trials were assessed according to the Newcastle–Ottawa scale [16]. Any disagreement was discussed until it was resolved by consensus.

### 2.6. Statistical Analysis

Descriptive statistic (mean and standard deviation) was used. Moreover, linear and angular deviations from the included study were analyzed in order to find any possible correlation with related scatter plots (MedCalc, MedCalc Software Ltd., Ostend, Belgium).

## 3. Results

### 3.1. Identified Articles

**Figure 2:**
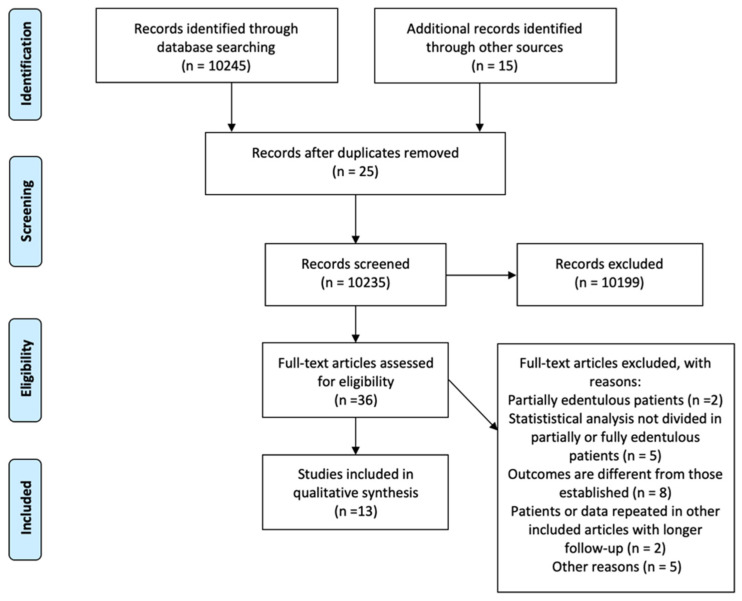
PRISMA flowchart.

The initial search resulted in 10,245 articles. Additional 15 articles were collected through manual screening. After title and abstract evaluation, a total of 36 studies were selected for full-text assessment. Twenty-two articles [17,18,19,20,21,22,23,24,25,26,27,28,29,30,31,32,33,34,35,36,37,38] were excluded after the full-text analysis (Table 1). A total of 13 studies [39,40,41,42,43,44,45,46,47,48,49,50,51] resulted eligible for qualitative analysis (Figure 2).

### 3.2. Quality Assessment

**Figure 3:**
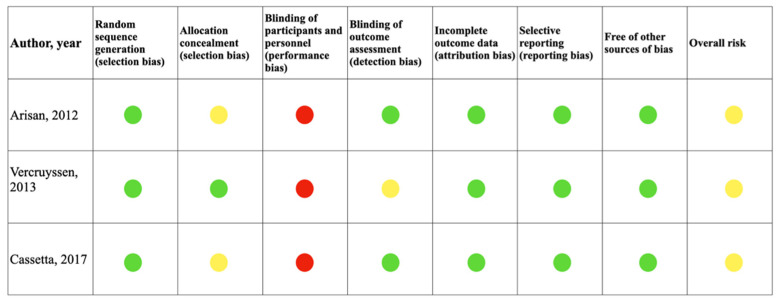
Quality assessment of the included studies following the Cochrane Collaboration’s tool 2 for assessing risk of bias in randomized trials (green, low risk; yellow, unclear risk; red, high risk).

**Figure 4:**
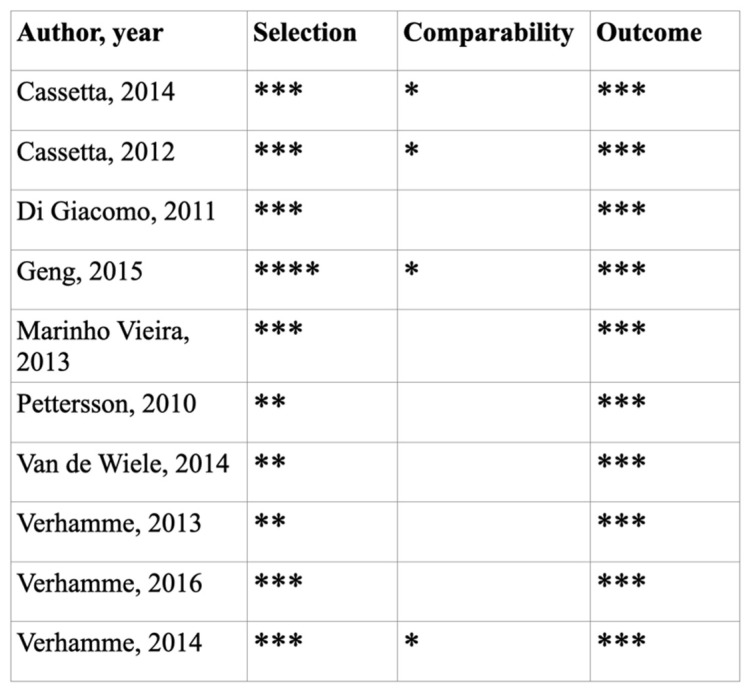
Quality assessment of the included studies following the Newcastle–Ottawa Scale for assessing the quality of nonrandomized trials. (Good quality: 3 or 4 *; Fair quality: 2 *; Poor quality: 0 or 1 *).

The results of the risk of bias assessment for the included RCTs according to the recommendations of the Cochrane Handbook for Systematic Reviews of Interventions [15] and of the prospective clinical trials assessed according to the Newcastle–Ottawa scale [16] are summarized in Figure 3 and Figure 4.

### 3.3. Included Studies

A total of 10 studies [40,42,43,44,45,46,47,48,49,50] were classified as prospective studies and 3 were RCTs [39,41,51]. The main characteristics of the included studies were summarized in Table 2.

**Figure 5:**
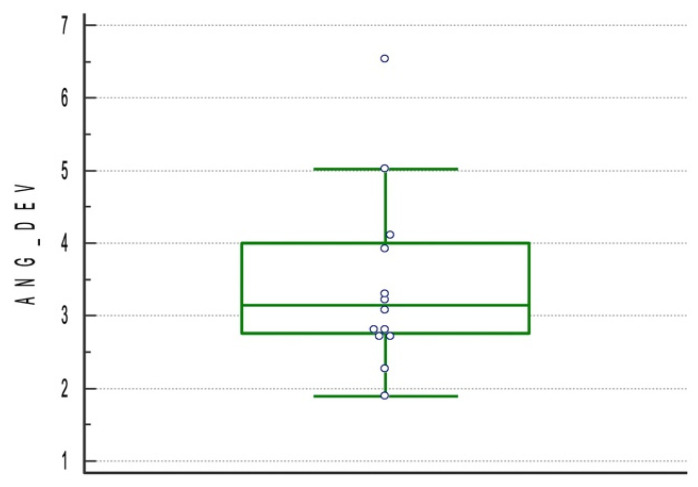
Boxplot for angular deviation of the included studies.

A total of 1556 implants were placed in 277 edentulous patients using a mucosa-supported template with flapless surgery. In one RCT [39], patients were randomly treated by experienced (at least 500 implants inserted using computer-guided implantology) or inexperienced (no experience in computer-guided surgery, but at least 500 implants inserted free-hand) surgeons. In two studies [49,50], the accuracy data were taken only from the fixed-template group. In one study [51], implants were randomly planned after computer tomography (CT) scan or after CBCT scan. In one RCT [34], patients were randomly treated with two different types of mucosa-supported templates. Six different accuracy evaluation softwires were used in the included studies. The NobelGuide Validation System (Nobel Biocare AG, Kloten, Switzerland) was used in five studies [42,43,44,46,47] to evaluate the accuracy of 527 implants. Mimics (Materialise NV, Leuven, Belgium) was used in four studies [41,45,49,50] to evaluate the accuracy of 686 implants. Simplant (DentsplySirona, York, PA, USA) was used in one study [48] to evaluate 111 implants. Analyze (AnalyzeDirect, Lenexa, KS, USA) was used in one study [51] to evaluate 102 implants. GeomagicStudio (3DSystems, Rock Hill, SC, USA) was used in one study [39] to evaluate 70 implants. Rhino 4.0 (Rhinoceros, Robert McNeel & Associates, Seattle, WA, USA) was used in one study [40] to evaluate 60 implants. The resulted angular deviation of the included studies was 3.42° ± 1.13° (95% CI 2.82–4.03) (Figure 5).

**Figure 6:**
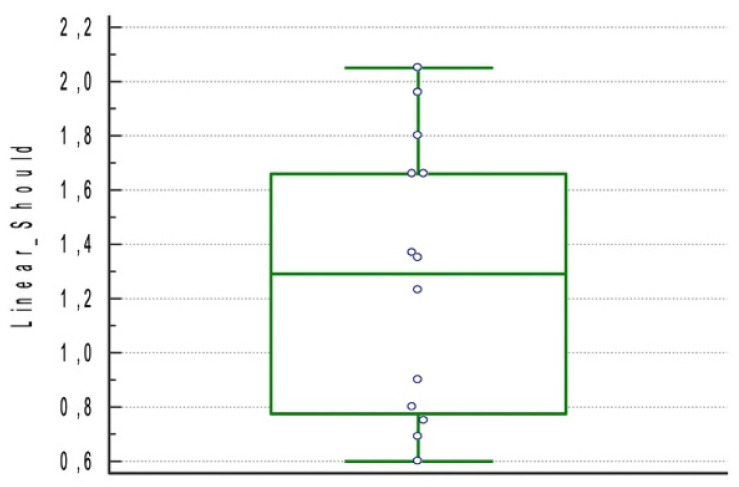
Boxplot for linear deviation at shoulder point of the included studies.

The resulted linear deviation at shoulder point was 1.23 mm ± 0.49 mm (95% CI 0.97–1.49) (Figure 6).

**Figure 7:**
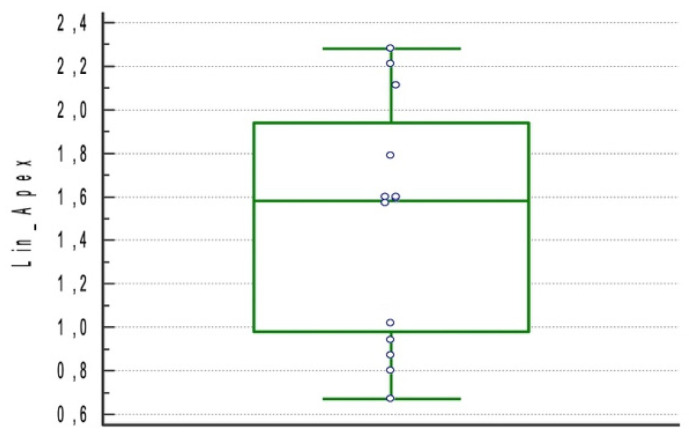
Boxplot for linear deviation at apex point of the included studies.

The resulted linear deviation at apex point was 1.46 mm ± 0.54 mm (95% CI 1.17–1.74) (Figure 7).

**Figure 8:**
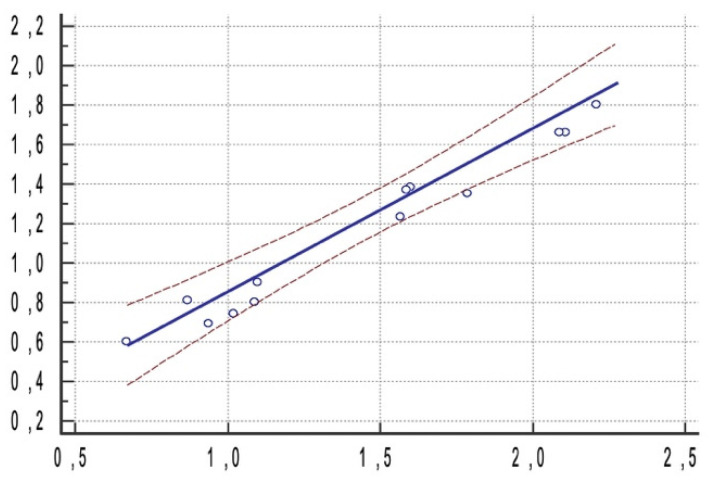
Regression equation (y = 0.03080 + 0.8254x, blue line) and confidence intervals (brown lines) assessing a statistically significant correlation between linear deviation at apex point and linear deviation at shoulder point.

No statistically significant correlations were found between linear (at shoulder and at apex points) deviations and angular deviations (correlation coefficient 0.35 and 0.27, respectively). A statistically significant correlation was found between linear deviations at shoulder and at apex point (correlation coefficient 0.91) that can be summarized by the regression equation y = 0.03080 + 0.8254x (Figure 8).

## 4. Discussion

The aim of this systematic review was to assess the accuracy of implant placement by means of static template mucosa-supported computer-guided flapless surgery in complete-arch rehabilitations. Thirteen studies were included in the review and the accuracy data of angular and linear deviations were analyzed. The quality assessment was performed following the Cochrane Collaboration’s tool for assessing risk of bias in RCTs and the Newcastle–Ottawa scale for prospective studies providing a high-quality statement on the results and none of the included studies reported high risk of bias (Figure 2 and Figure 3).

However, to the best of our knowledge, this is the first study reviewing the literature concerning the accuracy of implant placement by means of static template mucosa-supported computer-guided flapless surgery in complete-arch rehabilitations. In this study, a total of 1556 implants were analyzed concerning linear and angular deviations in order to assess linear and angular deviations. Recent systematic review by Seo and Juodzbalys [52] aimed to assess how accurate was implant placement with mucosa-supported templates in edentulous patients. The values reported were a range of expected deviation according to the available literature (0.67–2.19 mm, 0.6–1.68 mm and 2.6° to 4.67° for apical, coronal and angular deviation, respectively). The present systematic review goes one step beyond as the reported values of deviation of 1556 implants placed flapless were analyzed and summarized in mean values ± SD. In a systematic review by Tahmaseb et al. [9], the reported values for angular deviation were 3.3° and 1.3 mm at shoulder point and 1.5 mm at apex point for linear deviations in complete edentulous patients. These findings are in accordance with the results of the present systematic review, providing high-accuracy of static computer-aided surgery in complete-arch restorations. The standard deviations reported in this study (±1.13° angular deviation, ±0.49 mm linear deviation at shoulder point and ± 0.54 mm linear deviation at apex point) confirm that a 2-mm margin of safety needs to be applied when implant position is planned, as reported in the previously published systematic review by Thamaseb et al. [9], in order to avoid contacts or injuries to near anatomical structures. The computer-guided assisted implant insertion provided a more comprehensive and reliable treatment approach, enhancing clinical and aesthetic patient-related outcomes when compared to the free-hand surgery [53]. A recent systematic review by Tattan et al. [54] reported significantly lower angular, coronal and apical deviation values for implants placed by means of static-computer-guided surgery compared to free-hand implant placement.

Another systematic review by Gallardo et al. [10] reported that mucosa-supported templates could be influenced in the seating by keratinized mucosa thickness, local anesthesia and width of the alveolar bone. These factors could badly impact the accuracy of implant placement, increasing the risk of damages to the anatomical structures near the implant’s recipient sites. Moreover, the template fixation procedure is crucial to transfer the correct implant coordinates from the digital planning to the patient’s mouth. The properly seating of the surgical templates is based on the intaglio surface, which must be well replicated from the diagnostic template, in order to reproduce the same position that has been used to plan the implants in the planning software. Anchor pins are widely used to fix the surgical templates, and they need to be properly planned in sufficient amount of bone and in strategical positions that give stability to the templates.

Moreover, the present study aimed to assess if there was any statistically significant correlation between linear deviation at shoulder point, linear deviation at apex points and angular deviation. No statistically significant correlations were found between linear (at shoulder and at apex points) deviations and angular deviations (correlation coefficient 0.35 and 0.27, respectively). A statistically significant correlation was found between linear deviations at shoulder and at apex point (correlation coefficient 0.91). The correlation may be justified by the intrinsic surgical technique. When a fully guided implant surgery is performed, a surgical template with fixed sleeves and reducers guides the drill’s pathway through the bone recipient site and the surgeon cannot change or control the drill’s pathway while it is in function, unless a free-hand surgery is performed. The sleeves and the reducers are positioned above the implant recipient site, influencing the overall drill pathway from the insertion to the apex points. Static computer-assisted implant positioning is often associated with a flapless surgery, reducing surgical trauma and consequently postoperative pain and swelling, and patient’s discomfort [53,55]. In the included studies, all the 1556 implants were placed flapless. However, several factors can influence flapless surgery. The overall digital data acquisition workflow needs to be well conducted, the diagnostic template had to be properly relined on the edentulous arches and had to be properly worn by the patients while the CBCT examination was performed. Moreover, the analogic or digital impressions must be properly evaluated [56]. These steps should be performed after receiving a well conducted training by experienced clinicians to avoid bad positioning of the implants [39,45,52].

Because it was designed as a systematic literature review, the main limitation of this study is intrinsic in the quality of the included studies. Moreover, while the flapless approach in complete arch rehabilitations is widely approved, there are still missing data on its overall accuracy when computer guided surgery is performed.

## 5. Conclusions

Computer-assisted flapless implant placement by means of mucosa-supported templates in complete-arch restorations can be considered a reliable and predictable treatment choice, despite the potential effects that a flapless approach could bring to the overall treatment. Deviations from pre-planned implant coordinates may be the results of several errors in the overall planning workflow, leading to an inaccurate implant placement which may negatively influence aesthetic and functional results. In addition, RCTs and prospective studies comparing static and dynamic guided implant placement accuracy in complete arch rehabilitations have one single workflow that could lead to very low risk of bias in each study.

## Figures and Tables

**Table 1:** Excluded studies and reason for exclusion.

Excluded Study	Reason for Exclusion
Amorfini et al., 2016 [17]; Testori et al., 2014 [18].	The study analyzed partially edentulous patients.
Meloni et al., 2015 [19]; Tallarico et al., 2018 [20]; Pozzi et al., 2014 [21]; Cassetta et al., 2011 [22]; Cassetta et al., 2011 [23].	Results or statistical analysis are comprehensive and not divided in partially or fully edentulous patients.
Bernard et al., 2018 [24]; Tallarico et al., 2015 [25]; Pomares et al., 2010 [26]; Landàzuri-Del Barrio et al., 2011 [27]; Meloni et al., 2010 [28]; Orentlicher et al., 2014 [29]; Marra et al., 2017 [30]; Katsoulis 2011 [31].	The outcomes are different from those established.
Vasak et al., 2011 [32].	The authors do not report the number of implants placed in total and in partial edentulous patients.
Vercruyssen et al., 2014 [33]; Vercruyssen et al., 2014 [34].	Patients or data repeated in other included articles with longer follow-up.
Beretta et al., 2014 [35].	The sample is too small.
El Kholy et al., 2018 [36].	In vitro study.
Stübinger et al., 2014 [37].	The study analyzed bone-supported templates.

**Table 2:** Main characteristics of the included studies.

Author, Year	Study Design	Study Group	N° of Implants Evaluated	Evaluation Software	Angular Deviation ± SD (°)	Linear Deviation at Shoulder Point ± SD (in mm)	Linear Deviation at Apex Point ± SD
Petterson, 2010 [46]	Prospective	1	139	NobelGuide Evaluation Software	2.26 ± 2.53	0.80 ± 0.89	1.09 ± 1.18
Di Giacomo, 2012 [40]	Prospective	1	60	Rhino 4.0	6.53 ± 4.31	1.35 ± 0.65	1.79 ± 1.01
Cassetta, 2012 [50]	Retrospective	Fixed	66	Mimics	4.10 ± 2.43	1.66 ± 0.57	2.11 ± 2.22
Arisan, 2012 [51]	RCT	CT	50	Analyze	3.3 ± 1.08	0.75 ± 0.32	0.80 ± 0.35
Arisan, 2012 [51]		CBCT	52		3.47 ± 1.14	0.81 ± 0.32	0.87 ± 0.32
Marinho Vieria, 2013 [47]	Prospective	1	62	NobelGuide Evaluation Software	1.89	1.80	2.21
Verhamme, 2014 [42]	Prospective	1	104	NobelGuide Evaluation Software	2.81 ± 0.36	1.37 ± 0.17	1.59 ± 0.18
Vercruyssen, 2014 [41]	RCT	MATMU	55	Mimics	2.86 ± 1.16	1.23 ± 0.60	1.57 ± 0.71
Vercruyssen, 2014 [41]		FAMU	52		2.71 ± 1.36	1.38 ± 0.64	1.60 ± 0.70
Cassetta, 2014 [49]	Prospective	Fixed	144	Mimics	1.09 ± 2.40	1.66 ± 0.58	2.09 ± 0.75
Van de Wiele, 2014 [45]	Prospective	1	75	Mimics	5.02 ± 0.39	2.05 ± 0.14	1.60 ± 0.14
Cassetta, 2017 [39]	RCT	Inexperienced	33	GeomagicStudio	3.21	0.60 ± 0.25	0.67 ± 0.34
Cassetta, 2017 [39]	RCT	Experienced	37	GeomagicStudio	3.07	0.74 ± 0.18	1.02 ± 0.44
Verhamme, 2015 [44]	Prospective	1	150	NobelGuide Evaluation Software	3.92 ± 0.81	1.96 ± 0.45	2.28 ± 0.52
Geng, 2015 [48]	Prospective	1	59	Simplant	2.71 ± 2.58	0.69 ± 0.66	0.94 ± 0.75

## Data Availability

Not applicable.

## Supplementary Materials

The following supporting information can be downloaded at: https://www.mdpi.com/article/10.3390/ma15041462/s1.
