# Supplementary material for: Accuracy of Computer-Assisted Flapless Implant Placement by Means of Mucosa-Supported Templates in Complete-Arch Restorations: A Systematic Review
Source: Materials (Basel). 2022 Feb 16;15(4):1462. doi: 10.3390/ma15041462 (PMC8880344; doi:10.3390/ma15041462)
Supplement: Supplementary file 1 [file materials-15-01462-s001.zip › materials-1591806-supplementary.pdf]

EDENTULOUS

CassettaM,PompaG,DiCarloS,PiccoliL,PacificiA,PacificiL. The influence of smoking and surgical technique on the accuracy of mucosa- supported stereolithographic surgical guide in complete edentulous upper jaws. Eur Rev Med Pharmacol Sci 2012;16:1546–1553 ESCLUSO PERCHE' EDENTULIA TOTALE **PMID: 23111968**

Pettersson A, Komiyama A, Hultin M, Nässtrôm K, Klinge B. Accuracy of virtually planned and template guided implant surgery on edentate patients. Clin Implant Dent Relat Res 2012;14:527–537. ESCLUSO PERCHE' E' SU EDENTULI **PMID: 20491812**

Arisan, V., Karabuda, C.Z. & Ozdemir, T. (2010) Implant surgery using bone- and mucosa- supported stereolithographic guides in totally edentulous jaws: surgical and post-operative outcomes of computer-aided vs. standard techniques. Clinical Oral Implants Research 21: 980–988 **ESCLUSONPERCHE' EDENTULIA TOTALE. PMID: 20497439**

Gillot, L., Noharet, R. & Cannas, B. (2010) Guided surgery and presurgical prosthesis: preliminary results of 33 fully edentulous maxillae treated in accordance with the NobelGuide protocol. Clinical Implant Dentistry and Related Research 12 (Suppl 1): e104–e113 **ESCLUSO PERCHE' EDENTULIA TOTALE. PMID: 20455905 NO 2008**

Komiyama, A., Hultin, M., Na¨sstro¨m, K., Benchimol, D. & Klinge, B. (2012) Soft tissue conditions and marginal bone changes around immediately loaded implants inserted in edentate jaws following computer guided treatment planning and flapless surgery: A >/=1-year clinical follow-up study. Clinical Implant Dentistry and Related Research 14: 157–169. **ESCLUSO PERCHE' SU EDENTULI. PMID: 19793330 NO 2009**

Meloni, SM., De Riu, G., Pisano, M., Cattina, G. & Tullio, A. (2010) Implant treatment software planning and guided flapless surgery with imme- diate provisional prosthesis delivery in the fully edentulous maxilla. A retrospective analysis of 15 consecutively treated patients. European Journal of Oral Implantology 3: 245–251. **ESCLUSO PERCHE' EDENTULIA TOTALE. PMID: 20847994**

Pomares, C. (2010) A retrospective study of edentulous patients rehabilitated according to the 'all- on-four 'or the 'all-on-six ' immediate function concept using flapless computer-guided implant surgery. European Journal of Oral Implantology 3: 155–163. **ESCLUSO PERCHE' COMPLETAMENTE EDENTULI PMID: 20623040**

D'Haese J, Van De Velde T, Elaut L, De Bruyn H. A prospective study on the accuracy of mucosally supported stereolithographic surgical guides in fully edentulous maxillae. Clin Implant Dent Relat Res 2012;14:293–303. **ESCLUSO PERCHE' EDENTULIA TOTALE. PMID: 19906267 NO 2009**

Soares MM, Harari ND, Cardoso ES, Manso MC, Conz MB, Vidigal GM, Jr. An in vitro model to evaluate the accuracy of guided surgery systems. Int J Oral Maxillofac Implants 2012;27:824–831. **ESCLUSO PERCHE' IN VITRO**

Pettersson A, Kero T, Gillot L, et al. Accuracy of CAD/CAM-guided surgical template implant surgery on human cadavers: Part I.
J Prosthet Dent 2010;103:334–342. **ESCLUSO PERCHE' SU CADAVERI**

Tahmaseb A, van de Weijden JJ, Mercelis P, De Clerck R, Wismeijer D. Parameters of passive fit using a new technique to mill implant- supported superstructures: An in vitro study of a novel three-dimensional force measurement-misfit method. Int J Oral Maxillofac Implants 2010;25:247–257. **ESCLUSO PERCHE' IN VITRO**

Cassetta, M., Giansanti, M., Di Mambro, A., & Stefanelli, L. V. (2014). Accuracy of positioning of implants inserted using a mucosa- supported stereolithographic surgical guide in the edentulous maxilla and mandible.

International Journal of Oral and Maxillofacial Implants, *29*(5), 1071–1078. https://doi.org/10.11607/jomi.3329 **ESCLUSO PERCHE' EDENTULI. PMID: 25216132**

D'haese, J., Van De Velde, T., Elaut, L., & De Bruyn, H. (2012). A prospective study on the accuracy of mucosally supported stereolithographic surgical guides in fully edentulous maxilla. *Clinical Implant Dentistry and Related Research*, *14*(2), 293–303. https://doi.org/10.11 11/j.1708-8208.2009.00255 **ESCLUSO PERCHE' EDENTULI PMID: 19906267 NO 2009**

Vieira, D. M., Sotto-Maior, B. S., Barros, C. A., Reis, E. S., & Francischone, C. E. (2013). Clinical accuracy of flapless computer-guided surgery for implant placement in edentulous arches. *The International Journal of Oral & Maxillofacial Implants*, *28*(5), 1347–1351. https://doi. org/10.11607/jomi.3156 **ESCLUSO PERCHE' EDENTULI PMID: 24066327**

Verhamme LM, Meijer GJ, Boumans T, de Haan AF, Berge SJ, Maal TJ. A clinically relevant accuracy study of computer- planned implant placement in the edentulous maxilla using mucosa-supported surgical templates. Clin Implant Dent Relat Res 2015;17:343-52. **ESCLUSO PERCHE' TOTALMENTE EDENTULO. PMID: 23879524**

Meloni SM, De Riu G, Pisano M, Cattina G, Tullio A. Implant treatment software planning and guided flapless surgery with immediate provisional prosthesis delivery in the fully edentulous maxilla. A retrospective analysis of 15 consecutively treated patients. Eur J Oral Implantol 2010;3:245–251 **ESCLUSO PERCHE' EDENTULO PMID: 20847994 DOPPIONE**

39. Pomares C. A retrospective study of edentulous patients rehabilitated according to the 'all-on-four' or the 'all-on-six' immediate function concept using flapless computer-guided implant surgery. Eur J Oral Implantol 2010;3:155–163. **ESCLUSO PERCHE' EDENTULO. PMID: 20623040 DOPPIONE**

41. Landázuri-Del Barrio RA, Cosyn J, De Paula WN, De Bruyn H, Marcantonio E Jr. A prospective study on implants installed with flapless-guided surgery using the all-on-four concept in the mandible. Clin Oral Implants Res 2013;24:428–433. **ESCLUSO PERCHE' EDENTULO. PMID: 22092825**

33. Cannizzaro G, Torchio C, Leone M, et al. Immediate versus early loading of flapless-placed implants supporting maxillary full-arch prostheses: a randomised controlled clinical trial. Eur J Oral Implantol 2008;1:127–39. **EDENTULI PMID: 20467650 NO**

SINGOLI, CONTROLLARE SE C'E' QUALCHE EDENTULO:

21443593 EDENTULI PARZIALI

20447264 EDENTULI PARZIALI

21244498 ENTRAMBI E LA STATISTICA E' DIVISA (VASAK 2011)

22616060 NOT FOUND

24460748 statistica unita ma divide bene supporto osseo e mucoso!!!

21819249 edentuli totali

25177520 edentuli totali (guarda risultati non criteri di inclusione)

25181255 totalmente edentuli

25318961 totalmente edentuli

21745330 forse è ripetuto per ora va bene, poi cerchiamo il clone

19939691 partially edentulous

19438944 partially edentulous

25197015 no abbiamo follow up più lungo

25237668 no statistica mista

23062140 consensus

26385623 review

22984962 statistica mista

25346154 single tooth

23356732 rialzo di seno

22092586 partially edentulous

18672982 no è del 2008

20231051 partially edentulous

26652644 single tooth

26119019 no in vitro

25985057 no technical report

25835793 no statistica mista

22050241 case report single

22976712 no case report single

20589261 no navigation

25198877 statistica mista

23062136 revisione

20711144 no non specifica follow up

20967309 no 6 pazienti

19220842 statistica mista

22001378 doppione

21414133 ok totali

20128828 NO 2009

20074239 no in vitro

22172094 partially 5 pazienti

22235792 doppione

21841978 no statistica unita

22092586 ok totali

22551385 no in vitro

21959426 partially edentulous

22789635 doppione

23593625 doppione statistica non chiara

26309497 misto ma fanno differenza nei risultati

24353883 risultati non specificati tra parziale e totale

20491812 ok totali

26972545 partially o single

23167722 ok totali

25318961 DOPPIONE

25179585 c’è già follow up più lungo

27887876 ok totali

24066327 DOPPIONE

23527361 partially edentulous

22789635 no per statistica

20626424 parzialmente edentulous

22616058 doppione

23057043 NO

29543930 no in vitro

23377065 no impianti pterogomascellari

25199032 edentulo https://www.oralsurgeryny.com/files/2013/01/CSR-COMPENDIUM-FINAL-9-14.pdf

22616058 no per statistica unita doppione

20728034 no guidata

22043463 no guidata

19744199 no guidata

22140661 no guidata

22819332 no guidata

23347348 no guidata

20728034 NO

22235792 (parte 2) edentulo

64. Abboud M, Wahl G, Guirado JLC, et al. CT guided implant surgery with immediate loading: comparison and outcomes of two technologies. Int J Oral Maxillofac Implants 2012;27(3):634–43. NON LO TROVO SU PUBMED
